# Prevalence and Possible Role of *Candida* Species in Patients with Psoriasis: A Systematic Review and Meta-Analysis

**DOI:** 10.1155/2018/9602362

**Published:** 2018-05-06

**Authors:** Aldona Pietrzak, Ewelina Grywalska, Mateusz Socha, Jacek Roliński, Kinga Franciszkiewicz-Pietrzak, Lidia Rudnicka, Marcin Rudzki, Dorota Krasowska

**Affiliations:** ^1^Department of Dermatology, Venereology and Pediatric Dermatology, Medical University of Lublin, Ul. Radziwillowska 13, 20-080 Lublin, Poland; ^2^Department of Clinical Immunology and Immunotherapy, Medical University of Lublin, Ul. Chodźki 4a, 20-093 Lublin, Poland; ^3^Department of Internal Medicine and Cardiology, First Military Clinical Hospital with the Outpatient Clinic, Al. Racławickie 23, 20-048 Lublin, Poland; ^4^Department of Surgical Oncology, Medical University of Lublin, Ul. Staszica 11, 20-081 Lublin, Poland; ^5^Department of Dermatology, Medical University of Warsaw, Ul. Koszykowa 82a, 02-008 Warsaw, Poland; ^6^Chair and Department of Jaw Orthopaedics, Medical University of Lublin, Ul. Karmelicka 7, 20-081 Lublin, Poland

## Abstract

Although fungal colonization is implicated in the pathogenesis of psoriasis, its prevalence remains unclear. The aim of this systematic review and meta-analysis was to provide an overview on the prevalence of *Candida* species in patients with psoriasis. We searched databases (MEDLINE, EMBASE, Cochrane Central Register of Controlled Trials, and http://clinicaltrials.gov) to identify studies involving subjects of any age with an established diagnosis of psoriasis and healthy controls, who were tested for carriage of *Candida* spp. on the skin or mucosal membranes (or saliva and stool), or presented with clinical candidiasis with microbiologically confirmed etiology. We identified nine cross-sectional studies including a total of 1038 subjects with psoriasis (psoriatics) and 669 controls. We found *Candida* species detection rates for psoriatics were significantly higher than those in the controls, especially in the oral mucosa milieux. These results suggest psoriasis may be one of the systemic diseases that predispose to oral *Candida* spp. carriage and infection.

## 1. Introduction

Psoriasis is a common chronic inflammatory disease of the skin and joint, affecting approximately 2–4% of the general population [1]. Depending on the study population, the prevalence of psoriasis varies from 0.09% in the United Republic of Tanzania [2] to 11.4% in Norway [3].

Early concepts of the etiology of psoriasis focused primarily on keratinocyte hyperproliferation. However, dysregulation of the immune system is now recognized as a critical event in the pathogenesis of psoriasis. The inflammatory cascade of psoriasis involves complex interactions between keratinocytes, dendritic cells, neutrophils, and particularly T cells [4]. Psoriasis is now considered to be an organ-specific T cell-driven inflammatory disease, with interplay among Th1, Th9, Th17, Treg, and Th22 cells contributing to the development of the disease, although the nature of the antigen (autoantigen or microbial) that activates psoriatic T cells remains controversial [5, 6].

Over the past several years, the association between the microbiome and inflammatory skin diseases has been increasingly recognized [7]. It is hypothesized that microbial dysbiosis of the skin and mucosa could trigger an exaggerated immune response in a susceptible host, inducing a persistent inflammatory process associated with autoimmune disorders [8]. Several microorganisms (including bacteria, viruses, and fungi) were found to act as superantigens that activate specific T cells and initiate, exacerbate, and maintain chronic inflammation in skin disease [9]. This interconnection has been most thoroughly studied for *Staphylococcus aureus* skin colonization in atopic dermatitis and psoriasis [10, 11].

In addition to bacteria, fungi have also been implicated in stimulating skin-associated lymphoid tissue. The *Candida* species are a part of the normal human microbiota, frequently colonizing the skin and mucosal membranes of gastrointestinal and genitourinary tracts [12]. These common commensal organisms are capable of causing opportunistic infection following the disruption of the normal microbiome, a breach in the integrity of the mucocutaneous barrier, or immunodeficiency [13].

Although dysbiosis and the role of the microbiome in the pathogenesis of inflammatory skin diseases have been intensively investigated, fungal colonization or infection has received minimal attention. Therefore, the aim of our systematic review and meta-analysis was to provide an overview on the prevalence and colonization with *Candida* spp. in patients with psoriasis.

## 2. Methods

This meta-analysis was conducted in accordance with the Preferred Reporting Items for Systematic Reviews and Meta-Analyses (PRISMA) statement [14].

### 2.1. Search Strategy

Two researchers (Mateusz Socha and Kinga Franciszkiewicz-Pietrzak) performed independent, comprehensive searches of the following databases, from inception through May 20, 2017: MEDLINE (accessed by PubMed), EMBASE, Cochrane Central Register of Controlled Trials, and http://clinicaltrials.gov. The following search terms were used: “psoriasis,” “psoriatic,” “psoriatics,” “*Candida*,” “*Candida* spp.,” “*Candida albicans*,” “candidiasis,” “fungal infection,” “carrier,” “carriage,” “mycosis,” “oral mucosa,” “tongue mucosa,” “gut mucosa,” “saliva,” “stool,” “skin,” “dermal,” “swab,” “epidemiological study,” and “cross-sectional study” and combinations thereof. The search was limited to human studies but not restricted to any particular language or publication date. Reference lists from all available review articles were also searched manually. Database search by keywords gave 128 results; additionally, 6 records were found manually by reference lists. From the total number of 134 records, 7 occurred to be duplicates. Among 118 excluded articles in 76, the detection rate of *Candida* spp. was not studied, 30 were review articles, and 12 were case reports. Authors concluded that 9 articles were eligible for data extraction.

### 2.2. Study Selection

Published studies were considered eligible for the meta-analysis whenever they included subjects of any age with an established diagnosis of psoriasis and healthy controls, who were tested for carriage of *Candida* spp. on the skin or mucosal membranes (or saliva and stool), or presented with clinical candidiasis with microbiologically confirmed etiology.

**Figure 1:**
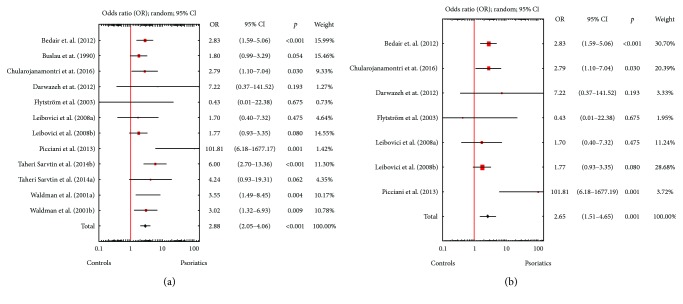
(a) Meta-analysis of nine studies (among them, three contained two different types of examined material) comparing odds ratios (ORs) for the detection of *Candida* spp. from the skin or mucosal membranes of psoriasis patients and healthy controls of any age. (b) Meta-analysis of six studies (among them, one contained two different types of examined material) comparing odds ratios (ORs) for the detection of *Candida* spp. from the skin or mucosal membranes of adult psoriasis patients and healthy controls.

To avoid double counting of patients included in more than one article by the same authors or research groups, patient recruitment periods were evaluated. Titles and abstracts of retrieved articles were independently evaluated by two researchers (Mateusz Socha and Kinga Franciszkiewicz-Pietrzak). Full-text articles were reviewed when abstracts did not provide sufficient information about inclusion and exclusion criteria. Full text of each article was reviewed independently, and its eligibility for inclusion in the meta-analysis was evaluated. Results were compared, and discrepancies were resolved through discussion and, if necessary, the inclusion of the third researcher (Ewelina Grywalska). The whole process is illustrated in Figure 1.

### 2.3. Primary Outcomes

The primary outcome of interest was a dichotomous variable: *Candida* spp. detection rate in psoriasis patients and healthy controls.

### 2.4. Data Extraction

Extracted data included information about the study design, characteristics and number of participants, source of examined material (skin versus mucosa), and *Candida* spp. detection rates. The details were summarized in standard extraction sheets, independently by two authors (Ewelina Grywalska and Jacek Roliński).

### 2.5. Statistical Analysis

Meta-analysis was conducted with Statistica 10 package (StatSoft, Tulsa, OK, USA), using the random effects model. The outcomes were calculated as odds ratios (OR) with their 95% confidence intervals (95% CIs). Homogeneity of the studies was verified with T2-test based on the weighted least square method. Post hoc sensitivity analysis was conducted to examine whether the overall finding was robust to an outlying study. In sensitivity analysis, sequential meta-analyses were repeated in which each study was excluded. Subgroup analyses were also carried out to determine *Candida* spp. detection rates from the skin and mucosa and/or in children and adults. The results of all tests were considered significant at *p* < 0.05.

## 3. Results

Of 134 records initially identified, titles and abstracts of 127 studies were assessed for eligibility after the exclusion of duplicates (Figure 1). A total of 118 records were excluded because the studies did not analyze *Candida* spp. detection rates (*n* = 76), or the retrieved publications were review articles (*n* = 30) or case reports (*n* = 12). Eventually, nine cross-sectional studies including a total of 1038 psoriatics and 669 controls [15–23] were available for analysis.

### 3.1. Description of Included Studies

Six analyzed studies included only adults [17–21, 23], and two studies involved both adults and children [16, 22]. Authors of one study did not specify if their subjects were older than 18 years [15]. Six studies solely analyzed material from mucosal membranes [15, 16, 19–21, 23], one solely analyzed material from the skin [17], and two included both mucosal and skin swabs [18, 22]. Two studies were conducted in Israel [16, 18], two in Jordan [19, 20], and the remaining in Germany [15], Sweden [17], Brazil [21] Iran [22], and Thailand [23]. All studies included patients with established diagnosis of psoriasis, both women and men. Detailed characteristics of all included studies are presented in Table 1.

### 3.2. Effect of Intervention

**Figure 2:**
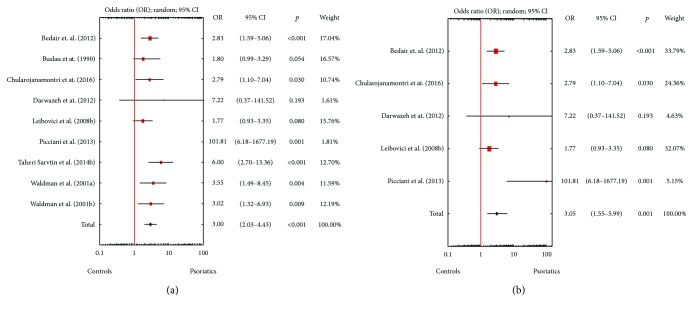
(a) Meta-analysis of eight studies (among them, one contained two different types of examined material) comparing odds ratios (ORs) for the detection of *Candida* spp. from mucosal membranes of psoriasis patients and healthy controls of any age. (b) Meta-analysis of five studies comparing odds ratios (ORs) for the detection of *Candida* spp. from mucosal membranes of adult psoriasis patients and healthy controls.

**Figure 3:**
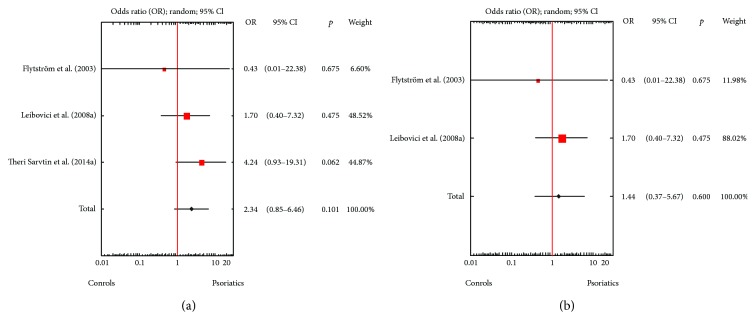
(a) Meta-analysis of three studies comparing odds ratios (ORs) for the detection of *Candida* spp. from the skin of psoriasis patients and healthy controls of any age. (b) Meta-analysis of two studies comparing odds ratios (ORs) for the detection of *Candida* spp. from the skin of adult psoriasis patients and healthy controls.

A random effects model pooling the results demonstrated that *Candida* spp. detection rates for psoriatics were significantly higher than those in the controls. The OR for isolation of *Candida* spp. from either the skin or the mucosal membranes of patients with psoriasis was 2.88 (95% CI: 2.05–4.06, *p* < 0.001), when both children and adults were included (Figure 1(a)), and 2.65 (95% CI: 1.51–4.65, *p* = 0.001) for solely adults (Figure 1(b)). Statistically significantly higher *Candida* spp. detection rates were also documented for mucosal membranes, either in all studies (OR = 3.00, 95% CI: 2.03-4.43, *p* < 0.001; Figure 2(a)) or solely in studies involving adults (OR = 3.05, 95% CI: 1.55–5.99, *p* = 0.001; Figure 2(b)). However, psoriasis patients and controls did not differ significantly in terms of *Candida* spp. isolation rates from the skin (Figures 3(a) and 3(b)). Visual inspection of the forest plot and statistical tests demonstrated considerable heterogeneity among studies. Sensitivity analysis (each study sequentially excluded) revealed that the result of the meta-analysis was not dependent on the outcome of any individual experiment (Table 2).

## 4. Discussion

To the best of our knowledge, this meta-analysis is the first to investigate the association between *Candida* spp. and psoriasis. The analysis was based on nine cross-sectional studies including a total of 1038 psoriatic patients and 669 healthy subjects. We found that the prevalence of *Candida* spp. colonization was significantly higher in patients with psoriasis compared with the control group.

The underlying mechanism for the association between psoriasis and *Candida* spp. has not yet been clearly identified. In healthy adults and children, the yeast-like fungi *Candida* spp. are not pathogenic and they are a commensal of the normal microbiome of the skin, oral cavity, gastrointestinal tract, and vaginal mucosa [13]. However, the abundance of *Candida* spp. on the skin may increase with altered immune function. Similarly to psoriasis, a higher prevalence of *Candida* colonization has been found in other inflammatory skin disorders, including atopic dermatitis [24].

Yeast-like fungi *Candida* play an important role in triggering psoriasis flares. *Candida* spp. antigens, especially *Candida albicans* surface proteins have been shown to have superantigen-like effects, resulting in the activation of T lymphocytes independently of antigen presentation and excessive release of proinflammatory cytokines [25, 26]. These cytokines, especially interleukin-23 (IL-23), promote the proliferation and survival of Th17 cells, which are essential for host defense against *C. albicans* [12]. In turn, Th17 cells release IL-17, which recruits neutrophils and contributes to *Candida* spp. clearance through releasing high amounts of antimicrobial peptides (AMPs), direct phagocytosis, and formation of neutrophil extracellular traps [27]. Finally, besides Th17 cells, skin resident and recruited Th9 cells bridge the innate and adaptive immune response against *C. albicans* infection [28].

The mechanisms involved in host defense against *C. albicans* share similar pathways associated with the pathogenesis of psoriasis. For example, it was recently shown that a Th9 cell subset, which secretes large quantities of IL-9, is increased in psoriatic skin lesions [6]. In addition, the IL-23/Th17/IL-17 pathway is one of the most important inflammatory processes in psoriasis, and its therapeutic blockade with biological agents is highly effective in the treatment of moderate to severe psoriasis [29]. However, at the same time, these pharmacological interventions affect antifungal immune responses and may partially promote increased prevalence of commensal colonization as well as the invasive growth of *C. albicans* [30, 31].

Subgroup analysis of our meta-analysis revealed significantly higher detection rates for *Candida* spp. on mucosal membranes. Recently available culture-independent methods of microorganisms profiling have improved our understanding of the microbiome and its impact on health [7]. So far, research has focused mainly on the skin microbiome, while data on the association between the gut microbiota and the skin disease is limited [32]. It is becoming increasingly apparent that gut microbiota might be able not only to regulate the local gastrointestinal immune system but also to affect the systemic immune system and thereby in turn influence other organs such as the skin [33]. From the evolutionary point of view, this interplay between the environmental microflora and the immune system at the barriers of the body is essential for educating the immune system and thus for our survival. However, in some genetically predisposed individuals, bacteria or fungi on the mucous membranes may lead to the activation of the local innate immune system and, in turn, induce an adaptive immune response [34]. These recent findings partially explain the presence of gastrointestinal symptoms in patients with psoriasis as well as coexistence with inflammatory bowel diseases [35, 36].

All analyzed studies in our meta-analysis indicated a higher prevalence of *Candida* spp. colonization in the oral cavity of psoriatic patients. These results suggest that psoriasis can be one of the systemic diseases that predispose to oral *Candida* spp. carriage and infection. Nevertheless, there are conflicting results concerning the association between oral candidiasis and systemic antipsoriatic treatment. In a study by Chularojanamontri et al. [23], the presence of oral *Candida* spp. infection assessed by culturing oral swabs was significantly higher in patients with psoriasis receiving immunosuppressive therapy. On the other hand, Bedair et al. [19] and Picciani et al. [21] showed a lack of this relationship. The methods used to confirm *Candida* spp. presence were concentrated oral rinse test and cytopathological examination, respectively. Discrepancies between studies may stem from difficulties in the differential diagnosis of oral psoriasis due to overlapping clinical and histological features with candidiasis. Candidiasis may clinically resemble psoriatic erythematous patches of the oral mucosa, and the two conditions share some similar features on histopathological examination such as hyperplastic rete ridges and intraepithelial neutrophils [37, 38]. Negative results of culture PAS stain of biopsy material or failure of antifungal treatment may help exclude *Candida* spp. etiology. In the view of the current results, further studies are needed to clarify the main factors predisposing patients with psoriasis for increased oral *Candida* spp. colonization and developing an active infection.

Surprisingly, in our study, psoriasis patients and controls did not differ significantly in the rate of *Candida* spp. isolated from the skin. There are two possible explanations of this observation. First, the results are based on the limited number of studies with considerable heterogeneity among them. Second, *C. albicans* growth could be inhibited by AMPs, which are excessively produced in a lesional psoriatic skin [39]. AMPs, also known as host defense proteins, are key molecules in the cutaneous innate immune system. They exhibit broad-spectrum killing activity against bacteria, fungi, and several parasites [40]. Calprotectin, a heterocomplex of the two calcium-binding proteins S100A8 and S100A9, is one of the most common skin-derived AMPs [41]. Calprotectin exerts fungistatic activity toward *C. albicans* [42]. It was also shown that calprotectin expression in the epidermis is upregulated in inflammatory skin diseases, such as psoriasis [43].

In conclusion, increased *Candida* colonization has been confirmed in subjects with psoriasis. This result is of great clinical importance due to the potential risk for *Candida* infections during treatment with novel biologic drugs such as IL-17 inhibitors, which significantly affect the antifungal immune response. Future studies are needed to investigate the interaction between *Candida* spp. colonization and immune system alterations in order to obtain possible new microbiome-targeted therapeutic options.

## Figures and Tables

**Table 1:** Characteristics of studies included in the meta-analysis.

Authors	Material	Age group	Study subjects (*n*)		Positive subjects (*n* (%))	
			Patients	Controls	Patients	Controls
Buslau et al. (1990)	Stool	Unspecified	343	50	233 (68%)	27 (54%)
Waldman et al. (2001a)	Saliva	Children and adults	50	50	39 (78%)	25 (50%)
Waldman et al. (2001b)	Stool	Children and adults	50	50	36 (72%)	23 (46%)
Flytström et al. (2003)	Axilla and groin	Adults	45	19	0 (0%)	0 (0%)
Leibovici et al. (2008a)	Axilla and groin	Adults	100	100	5 (5%)	3 (3%)
Leibovici et al. (2008b)	Tongue	Adults	100	100	32 (32%)	21 (21%)
Bedair et al. (2012)	Normal oral mucosa	Adults	100	100	69 (69%)	44 (44%)
Darwazeh et al. (2012)	Oral mucosal lesions	Adults	100	100	3 (3%)	0 (0%)
Picciani et al. (2013)	Normal oral mucosa	Adults	140	140	37 (26%)	0 (0%)
Taheri Sarvtin et al. (2014a)	Normal skin	Children and adults	100	50	15 (15%)	2 (4%)
Taheri Sarvtin et al. (2014b)	Normal oral mucosa	Children and adults	100	50	60 (60%)	10 (20%)
Chularojanamontri et al. (2016)	Normal oral mucosa	Adults	60	60	18 (30%)	8 (13.3%)

**Table 2:** Post hoc sensitivity analysis for studies included in the meta-analysis (each study excluded).

Excluded study	OR	SE	95% CI	*p*	Weight
Bedair et al. (2012)	2.94	0.61	1.96–4.43	<0.001	84.01%
Buslau et al. (1990)	3.14	0.59	2.17–4.53	<0.001	84.54%
Chularojanamontri et al. (2016)	2.93	0.57	2.00–4.29	<0.001	90.67%
Darwazeh et al. (2012)	2.86	0.52	2.01–4.07	<0.001	98.73%
Flytström et al. (2003)	2.93	0.52	2.07–4.14	<0.001	99.27%
Leibovici et al. (2008a)	2.98	0.55	2.07–4.28	<0.001	95.36%
Leibovici et al. (2008b)	3.13	0.59	2.17–4.51	<0.001	85.45%
Picciani et al. (2013)	2.66	0.35	2.05–3.45	<0.001	98.58%
Taheri Sarvtin et al. (2014a)	2.85	0.53	1.98–4.09	<0.001	95.65%
Taheri Sarvtin et al. (2014b)	2.57	0.42	1.87–3.53	<0.001	88.70%
Waldman et al. (2001a)	2.84	0.55	1.94–4.15	<0.001	89.83%
Waldman et al. (2001b)	2.90	0.57	1.97–4.27	<0.001	89.22%
Overall effect	2.88	0.50	2.05–4.06	<0.001	100.00%
